# Cognition in multiple sclerosis within the modern diagnostic and treatment era

**DOI:** 10.1093/brain/awaf446

**Published:** 2025-11-26

**Authors:** James F Sumowski, Sarah Levy, Ilana Katz Sand, Rachel Brandstadter, Emily Dvorak, Jordyn Anderson, Michelle T Fabian, Robin A Graney, Fred D Lublin, Aaron E Miller, Joshua Sandry

**Affiliations:** Corinne Goldsmith Dickinson Center for Multiple Sclerosis, Department of Neurology, Icahn School of Medicine at Mount Sinai, New York, NY 10029, USA; Corinne Goldsmith Dickinson Center for Multiple Sclerosis, Department of Neurology, Icahn School of Medicine at Mount Sinai, New York, NY 10029, USA; Corinne Goldsmith Dickinson Center for Multiple Sclerosis, Department of Neurology, Icahn School of Medicine at Mount Sinai, New York, NY 10029, USA; Department of Neurology, Perelman School of Medicine at the University of Pennsylvania, Philadelphia, PA 19104, USA; Department of Psychiatry, Weill-Cornell Medical College, New York, NY 10065, USA; Corinne Goldsmith Dickinson Center for Multiple Sclerosis, Department of Neurology, Icahn School of Medicine at Mount Sinai, New York, NY 10029, USA; Corinne Goldsmith Dickinson Center for Multiple Sclerosis, Department of Neurology, Icahn School of Medicine at Mount Sinai, New York, NY 10029, USA; Corinne Goldsmith Dickinson Center for Multiple Sclerosis, Department of Neurology, Icahn School of Medicine at Mount Sinai, New York, NY 10029, USA; Corinne Goldsmith Dickinson Center for Multiple Sclerosis, Department of Neurology, Icahn School of Medicine at Mount Sinai, New York, NY 10029, USA; Corinne Goldsmith Dickinson Center for Multiple Sclerosis, Department of Neurology, Icahn School of Medicine at Mount Sinai, New York, NY 10029, USA; Department of Psychology, Montclair State University, Montclair, NJ 07043, USA

**Keywords:** cognition, multiple sclerosis, memory, working memory, patient-reported outcome measures, processing speed

## Abstract

Slowed information processing speed has long been considered the principal cognitive deficit in multiple sclerosis, with slowed cognitive speed presumed responsible for downstream deficits in other functions, including memory. This speed-centric model was established over three decades ago before disease-modifying therapies were available, and contrasts with our current clinical experience, but nonetheless remains dominant and unquestioned. We re-evaluated this model among current patients with multiple sclerosis diagnosed and cared for within the modern diagnostic and treatment era (2001–25).

Within a case-control cohort, persons with early relapsing-remitting disease (≤5.0 years since diagnosis, *n* = 170) performed worse than neurologically-healthy controls (*n* = 45) on memory but not cognitive speed, and cognitive speed among patients remained normal and stable over the next 6 years despite subtle memory decline. Relative to four historical studies of early relapsing-remitting disease (diagnosed using older criteria), effect sizes for case-control differences in our cohort were much lower for cognitive speed, but comparable for memory.

An independent clinical cohort of 1004 consecutive patients aged 18–65 years with relapse-onset multiple sclerosis completed standard-of-care cognitive screenings between 2018 and 2025. We captured data from three independent periods. During the first period (*n* = 642), rates of poor performance (≤7.5th percentile) did not differ from normative expectations for cognitive speed (8.4%) or attention (9.0%), but were nonetheless elevated for verbal memory (23.8%) and visuospatial memory (14.8%). Patients during the second (*n* = 123) and third (*n* = 239) periods demonstrated memory deficits despite normal cognitive speed on co-normed tasks. Objective cognitive speed among current patients was remarkably similar to healthy normative expectations [*z*-score: mean (standard deviation), 0.03 (1.14); median (interquartile range), 0.00 (−0.67, 0.67)], and was much better than across several historical comparisons from 20–25 years ago. Self-reported cognitive deficits within the total clinical cohort versus control respondents indicated worst disease-related difficulties in expressive language (e.g. word-finding), followed by working memory and episodic memory, with a small difference in executive/speed that was fully explained by mood in relapsing-remitting disease. Current patient-reported attention/executive deficits were lower than 35 years ago, despite comparable memory difficulties. As an exception, attention/executive and cognitive speed deficits were observed in secondary-progressive disease.

The speed-centric model of cognitive dysfunction in multiple sclerosis is inaccurate within the modern diagnostic and treatment era. Memory deficits remain prevalent, and we highlight working memory maintenance as an important target for further investigation. The field requires testable models of memory dysfunction informed by contemporary cognitive neuroscience, with the goal of developing heretofore elusive treatments for memory deficits.

## Teaser

Sumowski *et al.* show that, in contrast to historical cohorts, contemporary patients with MS more often exhibit memory and word-finding difficulties than cognitive slowing. Because cognitive speed now appears largely preserved, the speed-centric model of cognitive dysfunction in MS may no longer reflect current disease patterns.

**See Leavitt (https://doi.org/10.1093/brain/awag107) for a scientific commentary on this article.**

## Introduction

**Figure 1:**
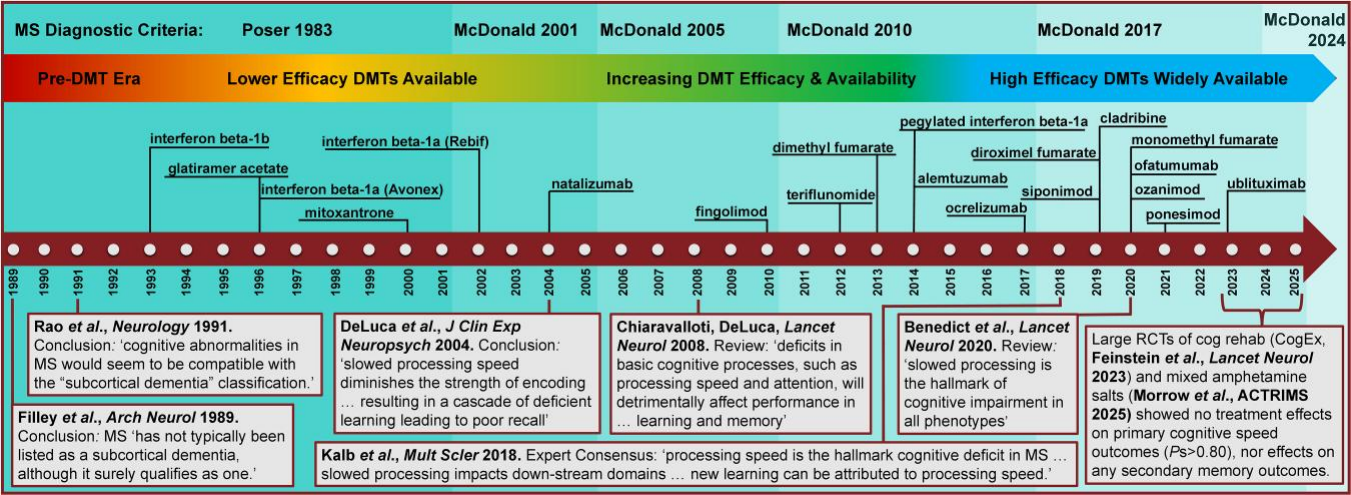
**Evolution of diagnostic criteria, treatment efficacy, and understanding of cognitive dysfunction in multiple sclerosis.** The speed-centric model of cognitive dysfunction in multiple sclerosis (MS) was established during the pre-disease-modifying treatment (DMT) era and when diagnostic criteria were less sensitive (Poser 1983 criteria^9^), but this model has remained dominant and unquestioned despite advances in treatment (DMTs listed by year of approval by the United States Federal Drug Administration) and diagnostic sensitivity (McDonald 2001,^10^ and subsequent revisions^11-13^ incorporating MRI evidence of MS pathology into diagnostic criteria). Despite dramatic advances in diagnostic sensitivity and DMTs, influential papers over three decades (e.g. see quoted text^2-4,6-8^) have sustained belief in slow processing speed as the hallmark cognitive deficit in MS, and the assumption that memory difficulties are the downstream consequence of slow cognitive speed. The speed-centric framework has not led to effective cognitive treatments; indeed, recent large-scale trials of cognitive rehabilitation plus physical exercise (CogEx)^14^ and mixed amphetamine salts^15^ have failed to improve primary cognitive speed and secondary memory outcomes. All patients in the current study were diagnosed in 2001 or later (i.e. McDonald criteria,^10-13^ potential access to DMTs at diagnosis), and were evaluated between 2016 and 2025. Further advancing diagnostic sensitivity, the latest revisions^16^ to the McDonald diagnostic criteria (in 2024) allow diagnosis of relapsing multiple sclerosis even without a symptomatic lesion if supported by adequate paraclinical evidence, with the hope that earlier diagnosis and treatment will lower risk for functional decline.

Foundational research conducted decades ago established the longstanding speed-centric model of cognitive dysfunction in multiple sclerosis,^1-5^ which posits slow cognitive speed as the principal deficit that leads to difficulties with memory and other functions.^6-8^ This model was rooted in an understanding of multiple sclerosis as a prototypical diffuse white matter disease, with cognitive dysfunction likened to subcortical dementia.^1,2^ As shown in Fig. 1, this framework was established before the first disease modifying therapy for multiple sclerosis became available in 1993, and research supporting this framework was conducted when lower efficacy injectable medications were the only therapeutic options. We now have numerous moderate and high efficacy therapies that protect against inflammatory lesion formation. Note also that early cognitive research was conducted when diagnostic criteria required more severe clinical presentations to support diagnoses.^9^ Incorporation of MRI evidence has improved diagnostic sensitivity,^10-13,16^ allowing earlier diagnosis and ascertainment of milder cases.^17-19^ These innovations help explain less physical disability among current patients.^20-22^ Understanding of multiple sclerosis pathophysiology has simultaneously undergone a paradigm shift emphasizing cortical pathology and neurodegeneration.^23-28^ Despite these advances, the speed-centric model of cognitive dysfunction has not evolved; it remains dominant and unquestioned,^7,8^ with authoritative literature continuing to highlight slow speed as the ‘hallmark cognitive deficit’ that encumbers downstream functions such as memory.^7^

The speed-centric model conflicts with our clinical experience; our patients are much more likely to report word-finding difficulty and memory problems than slowness of thinking. The speed-centric model appears perpetuated by a key psychometric misunderstanding, namely, that poor performance on the Symbol Digit Modalities Test (SDMT) indicates a processing speed deficit.^29-31^ With instructions to perform quickly, the SDMT has face validity as a cognitive speed metric; however, it was purposely designed as a high-sensitivity screener with multifarious demands to detect if any cognitive deficit exists (i.e. attention, speed, memory, language), not to isolate any specific deficit.^32,33^ Indeed, ample evidence documents memory contributions to SDMT in multiple sclerosis,^34-39^ including principal component analyses of influential cognitive batteries.^34-36^ Conversely, we cannot find research validating slow cognitive speed as the mechanism for poor SDMT in multiple sclerosis. Nonetheless, ‘SDMT’ and ‘processing speed’ have become synonymous (Rao *et al*.^31^), and the field has all but abandoned more parsimonious cognitive speed metrics,^30^ including within consensus batteries.^34,40^ This leaves us without empirical evidence for or against current validity of the model, which has implications for cognitive rehabilitation. Indeed, memory interventions remain elusive,^41^ and recent large-scale trials to improve cognitive speed have failed on their primary (SDMT) and secondary (memory) outcomes.^14,15^

Herein we reappraise the speed-centric model by examining cognitive speed and memory in relapse-onset multiple sclerosis diagnosed within the modern diagnostic and therapeutic era (2001–25). Cognitive speed is assessed with more parsimonious tasks than the SDMT, although SDMT is also reported for context and comparison to previous literature. First, we examine cognition in early relapsing-remitting disease versus controls in a research sample, and compare current case-control differences to those of historical case-control studies in early relapsing-remitting multiple sclerosis. We also examine cognitive change in our patient cohort over 3 to 6 years. Next, within a large independent clinical cohort, we investigate whether cognitive speed, memory, attention and executive function among current patients differs from healthy standardization samples, and whether cognitive speed among current patients differs from that reported in historical studies conducted before moderate or high efficacy treatments were available. We additionally examine self-reported difficulties across cognitive domains between patients with multiple sclerosis and demographically-matched controls, and assess whether self-reported deficits differ from those reported by patients three decades ago.

## Materials and methods

To maximize clarity and minimize redundancy, we first present all methods for the Reserve against Disability in Early Multiple Sclerosis (RADIEMS) case-control cohort, including performance metrics, historical comparisons and statistical analyses. We then present all methods for the clinical cohort. All group comparisons were two-tailed; *t*-tests were performed with bootstrapping (5000 samples). Significance was set at *P* < 0.050; corrections for multiple comparisons are described as necessary. Unless stated otherwise, values followed by a single number in parentheses [e.g. 12.5 (2.5)] are means and standard deviations (SD); values followed by two numbers in parentheses [e.g. 12.5 (10.0, 15.0)] are means and 95% confidence intervals. Unless stated otherwise, there were no concerns for skewness (≥1.0), kurtosis (≥3.0), or outliers [≥±3.0 × interquartile range (IQR)].

### RADIEMS case-control cohort

#### Participants

From October 2016 to December 2017, the RADIEMS study at Mount Sinai Hospital enrolled persons aged 20 to 50 years and diagnosed with relapsing-remitting multiple sclerosis for ≤5.0 years (McDonald 2010 criteria^12^) with no comorbid neurological or neurodevelopmental condition, serious mental illness (e.g. schizophrenia), or clinical relapse within the last 6 weeks. Control data were collected from neurologically-healthy friends and non-first-degree relatives of patients. Cognitive data were collected from patients and controls at baseline (Year 0) and Year 3 follow-up, and from patients at Year 6 follow-up.

#### Performance metrics

The Brief Repeatable Battery of Neuropsychological Tests (BRB)^42^ is a test battery developed in the 1990s for multiple sclerosis; it includes the SDMT, the Selective Reminding Test (SRT)^43,44^ to assess verbal memory, and was modified^45^ to include the Stroop paradigm requiring cognitive speed and executive control during rapid naming of ink colours of colour names printed in contrasting colour (e.g. the word ‘blue’ printed in red ink). RADIEMS participants completed the SDMT, SRT, and Stroop Color-Word Test (SCWT^46^), which allows direct comparisons to historical studies using the BRB. RADIEMS includes other non-BRB clinical and experimental tasks, but we limited analyses to SDMT, SRT and SCWT for parsimony and consistency with our aim of characterizing cognition in the modern era versus historical comparisons. Consistent with historical studies, we report scores for SDMT, SCWT (colour-word condition), and three SRT scores: long-term storage (LTS), consistent long-term retrieval (CLTR), delayed recall (DR). To place Year 0 scores on the same scale, raw scores were converted to *z*-scores using means and SD of controls.

#### Statistical analyses

##### Cross-sectional differences

We first assessed for differences between patients and controls in baseline age, sex, race/ethnicity, education, and estimated premorbid cognitive ability [Wechsler Test of Adult Reading (WTAR^47^)]. Raw scores were adjusted for variables with at least a trending difference between groups (*P* ≤ 0.10). Independent *t*-tests then assessed for differences between patients and controls in Year 0 cognitive test scores (Bonferroni-corrected *P* < 0.0167 for the three SRT scores).

##### Longitudinal change

To assess for attrition bias, we tested for differences in Year 0 demographic and clinical characteristics [including disability: Expanded Disability Status Scale (EDSS)] between participants who did versus did not return for follow-ups. Raw score change was derived for each task: (Year 3) − (Year 0), (Year 6) − (Year 0). Independent *t*-tests were used to assess for group differences in cognitive change from Year 0 to Year 3; one-sample *t*-tests assessed if cognitive change scores among patients differed from 0.0.

##### Historical comparisons

Literature review identified case-control studies^48-51^ in early relapsing-remitting multiple sclerosis (i) diagnosed for <5.0 years; with (ii) SDMT, Stroop, and SRT; and (iii) sample sizes ≥30 patients and ≥20 controls (≥0.80 power to detect large effects); and (iv) published >10 years ago. As minor exceptions, we included one study with a different but validated word-list learning task,^51^ and one study with patients <10 years since first symptom but mean <5.0 years since diagnosis.^50^ We calculated effect sizes (Hedge’s *g*) for case-control differences on each task for each study (effect size for SRT was averaged across LTS, CLTR and DR). Weighted mean effect sizes were calculated for each task across historical studies, which we compare to effect sizes for case-control differences in RADIEMS.

### Mount Sinai Hospital cognitive screening cohort

#### Retrospective chart review

The Corinne Goldsmith Dickinson Center for Multiple Sclerosis at Mount Sinai Hospital is a tertiary care centre with a catchment area encompassing the diverse New York Metropolitan Area. In August 2018, we established a clinic aiming to perform standard-of-care cognitive screenings for all patients. A retrospective chart review of this clinic captured clinical and cognitive data from all patients aged 18 to 65 years who were diagnosed with relapse-onset multiple sclerosis from 2001 to 2025 (McDonald criteria^10-13^). We excluded data from patients with other primary neurologic conditions (e.g. stroke), serious mental illness (e.g. schizophrenia) or relapse within 6 weeks.

#### Three independent and unique clinical datasets

**Figure 2:**
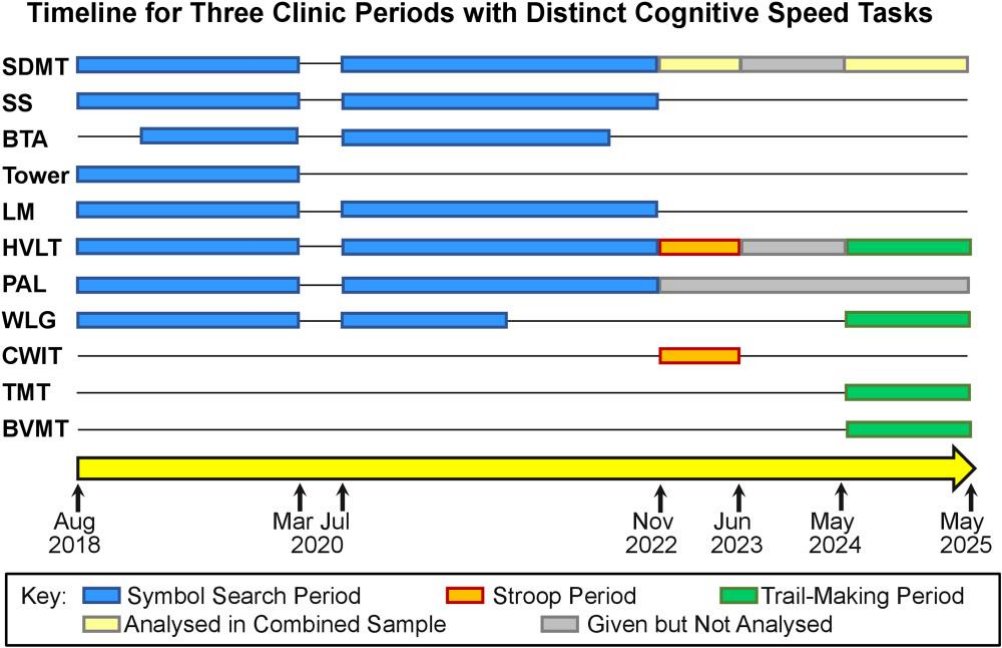
**Timelines for cognitive task periods in the clinical cohort.** Our core cognitive test battery evolves over time based on clinical judgment and research observations. Herein we analyse three independent datasets of unique patients from three periods with different cognitive speed tasks: Symbol Search (blue), Stroop (orange), and Trail-Making (green). Task names, abbreviations, and cognitive requirements are as follows: Symbol Digit Modalities Test^32^ (SDMT; state digits for as many symbols as possible across multiple rows in 90 s based on a key of nine digit-symbol pairings); Symbol Search^52^ (SS; identify visual target symbols among sets of distractors for as many trials as possible in 120 s; one of two tasks composing the Wechsler Processing Speed Index); Brief Test of Attention^53,54^ [BTA; maintain ongoing mental tally of digits (or letters) from strings of mixed digits and letters aurally-presented at rate of 1/s; total correct on 20 trials recorded]; Tower of London, 2nd Edition^55^ (Tower; plan and execute the most efficient approach to achieve target patterns; total moves and total time across 10 trials are recorded); Logical Memory-I^56^ (LM; immediately retell two aurally-presented vignettes, each presented for 25–30 s and each consisting of 25 discrete idea units); Hopkins Verbal Learning Test^57^ [HVLT; immediately recall 12 aurally-presented words on three learning trials; recall after 25 min; words recalled on learning trials (Total Learning, TL) and after delay (Delayed Recall, DR) recorded]; Paired Associate Learning^58^ (PAL; view locations of sequentially-presented abstract visual stimuli, then recall location when stimulus is represented; total errors across trials is recorded); Color Word Interference Test^59^ [CWIT; Stroop paradigm; rapid colour-naming, word-reading, and colour-word naming of ink colours with interference (i.e. the word ‘red’ printed in blue ink)]; Trail-Making Test^60^ (TMT; rapidly connect disseminated array of digits in numerical order (TMT-A), then digits and letters in alternating numerical and alphabetical orders (TMT-B)]; Word List Generation (WLG; state as many unique animals as possible is 60 s); Brief Visuospatial Memory Test^61^ [BVMT; study six figures in six locations, then draw as much of array as possible, on each of three learning trials (TL) and again after 25 min (DR)]. Note that tasks in green are co-normed,^62,63^ and tasks in orange are co-normed,^64^ which affords robust comparisons of within-patient performance across tasks. The yellow bar indicates that SDMT is not included within co-normative comparisons of that period, but raw and normative^32,65,66^ scores for SDMT are nonetheless captured and subsequently analysed within the combined clinical sample. Grey bars indicate that a task remained within our core battery, but either data were not captured from that period because cognitive speed was not assessed (June 2023 to May 2024), or that metric was not co-normed with tasks during orange and green periods (e.g. PAL).

We strive for a translational approach to cognition in multiple sclerosis, with research guided by clinical observations, and clinical work informed by research. We therefore periodically evaluate the sensitivity and utility of performance metrics within our cognitive test battery, and make adjustments accordingly. Figure 2 provides titles, abbreviations and descriptions for tasks in our core battery,^32,52-66^ and timelines for revisions. Current analyses use data captured from three non-overlapping periods distinguished by different core batteries, yielding three independent datasets named for the cognitive speed task of each period: Symbol Search, Stroop, Trail-Making. Our core battery approach ensured that all patients within a given period completed the same core tasks, thereby avoiding selection bias. Each period makes unique contributions to this investigation and strengthens conclusions via conceptual replication. The Symbol Search period provides the most data, includes a wider breadth of cognitive domains, and allows direct comparisons of current cognitive speed versus historical data on Symbol Search.^4^ The Stroop period benefits from co-normative comparisons between cognitive speed and memory, with comparisons to Stroop data from several historical case-control studies.^67^ The Trail-Making period permits robust within-subjects comparisons of cognitive speed, verbal memory, and visuospatial memory relative to the same healthy comparison group.^62,63^ Symbol Search, Stroop, and Trail-Making are the only established metrics of cognitive speed ever used in our clinic, although other tasks require rapid information processing or responses (e.g. Brief Test of Attention, Word List Generation). See Fig. 2 for titles and descriptions corresponding to task abbreviations used later.

#### Symbol search period

The battery contained Symbol Search, Hopkins Verbal Learning Test (HVLT-R), and paired associates learning (PAL) for all patients; Brief Test of Attention (BTA), Word List Generation (WLG), Tower, and LM-I were added or removed as shown (Fig. 2). Raw scores were converted to age-adjusted standard scores relative to healthy standardization samples for each task. Type of standard score differed across tests (e.g. *T*-score, scaled score); for uniformity, all were converted to z-scores corresponding to scaled scores [mean (SD) of 10 (3), range of 1–19, corresponding to *z*-scores ranging from −3.0 to 3.0 with 0.33 intervals].

A median split of time since diagnosis divided patients with relapsing-remitting disease into subgroups with shorter (early relapsing-remitting) and longer (later relapsing-remitting) disease durations. This allows more robust comparisons between secondary progressive and later relapsing-remitting multiple sclerosis because disease duration would be similar, and between early and later relapsing-remitting disease because phenotype is the same (cognitive performance was adjusted for age). Poor performance was defined as a scaled score ≤5, which has a *z*-score range of −1.835 (3.5th percentile) to −1.505 (7.5th percentile); normative rate of poor performance is therefore 7.5%. Logistic regressions test for differences in observed versus expected (7.5%) rates of poor performance across tasks for the total sample and subgroups (early relapsing-remitting, later relapsing-remitting, secondary progressive).

##### Historical comparisons

An influential 2004 publication^4^ reported rates of poor Symbol Search performance in relapsing-remitting and secondary-progressive disease. Patients were diagnosed using Poser 1983 criteria,^9^ and were evaluated before moderate or high efficacy therapies were available. Logistic regressions compare rates of poor performance on Symbol Search in our cohort versus the 2004 sample.^4^ Additionally, one-sample *t*-tests compare normative *z*-scores for Symbol Search in relapsing-remitting disease within our cohort versus mean performance reported in 2004,^4^ 2012,^68^ and 2022.^69^

#### Stroop period

Stroop tasks have been considered metrics of cognitive speed in multiple sclerosis due to equally poor performance across trials of rapid word-reading, colour-naming, and colour-naming during distraction.^67^ HVLT was co-normed^64^ with Color Word Interference Test (CWIT; our Stroop task), which bolsters comparison of cognitive speed and memory in our cohort relative to the same healthy standardization sample (*n* = 291). Raw scores were converted to age-adjusted scaled scores, then converted to *z*-scores. To derive more robust metrics (and reduce multiple comparisons), normative *z*-scores for HVLT (TL, DR) and CWIT trials were averaged into single memory and cognitive speed *z*-scores, respectively. Dependent *t*-tests and McNemar tests assessed for within-subjects differences in normative *z*-scores and rates of poor performance for cognitive speed and memory; norms convert to scaled scores (poor performance ≤7.5th percentile).

##### Historical comparisons

We derived effect sizes (*g*) for case-control differences in Stroop performance between multiple sclerosis (*n* = 464) and controls (*n* = 358) across 10 studies^5,70-76^ published from 1987 to 2008 (for a review, see Denny *et al*.^67^). Within our cohort, we first derived age-adjusted normative *z*-scores for CWIT Stroop trials (colour, word, colour-word) relative to the standardization sample of 650 healthy adults aged 18 to 65,^59^ then performed one-sample *t*-tests of differences between observed and expected (*z* = 0.0) performance; we compared effect sizes in our cohort to weighted mean effect sizes of historical studies.^5,70-76^

#### Trail-making period

Patients completed tasks of cognitive speed [Trail Making Test Part A (TMT-A), WLG, HVLT-TL, and visuospatial memory (BVMT-TL)] co-normed on 300 healthy persons aged 20–59 years (MATRICS)^62,63^; age range was therefore limited to 20–59 years. Raw scores were converted to age- and sex-adjusted normative *T*-scores, then converted to *z*-scores. Patients also completed TMT-B requiring cognitive speed and executive control (not co-normed within MATRICS, but meta-co-normative data^77^ used to compare TMT-A and TMT-B). Dependent *t*-tests and McNemar tests compared normative *z*-scores and rates of poor performance across co-normed^62^ TMT-A, WLG, HVLT and BVMT. MATRICS converts to *T*-scores (more granular than scaled scores; poor performance ≤5th percentile). Dependent *t*-tests assessed for within-subjects differences in normative *z*-scores^77^ for TMT-A and TMT-B, and independent *t*-tests assessed for differences between relapsing-remitting and secondary-progressive disease on TMT-A, TMT-B, and a contrast score isolating executive control [(TMT-B *z*-score) – (TMT-A *z*-score)].

#### Cognition in a combined clinical cohort

##### Cognitive speed

Normative *z*-scores were compiled across clinical periods: Symbol Search, CWIT colour-word, and TMT (mean *z* for TMT-A and TMT-B, converted to *z*-scores corresponding with scaled scores to match Symbol Search and CWIT). One-sample *t*-test examined whether the mean *z*-score differed from normative expectations (0.0).

##### Current versus previous SDMT performance

We compare SDMT raw scores in our combined clinical cohort to those reported for the 2006 validation sample (*n* = 291) of the Minimal Assessment of Cognitive Function in Multiple Sclerosis (MACFIMS).^34^ Current and MACFIMS datasets were both collected in New York State. Blind to all other information, we derived the largest possible subsample of current patients matching the 2006 sample for age and sex. We estimated premorbid ability with the WTAR^47^; although unreported in 2006, samples from the same clinic with similar characteristics had a normative *z*-score of 0.35 (0.57).^78-81^ We matched for premorbid ability if our cohort differed from this estimate. A one-sample *t*-test investigated whether current SDMT performance differed from mean performance in 2006. The 2006 paper derived *z*-scores relative to their control group (*n* = 56), and reported per cent of patients with *z*-scores < −1.5; we replicated their same procedures using their control group, and logistic regression compared rates of poor performance between current and previous samples.

#### Self-reported cognitive dysfunction

##### Multiple Sclerosis Cognitive Scale

The Multiple Sclerosis Cognitive Scale (MSCS)^82^ is an 8-item factor-analytically-derived self-report cognitive scale on which respondents indicate frequency of difficulties: never (0), rarely (1), sometimes (2), fairly often (3), very often (4). Excellent factor structure supports four MSCS subscales with good internal consistencies (α’s: 0.83 to 0.87) and test-retest reliabilities (intraclass correlations: 0.86 to 0.92): executive/speed (taking a long time to finish things, difficulty getting started even if there is lots to do), working memory (losing train of thought, forgetting why you entered a room), expressive language (word on the tip of your tongue, trouble clearly expressing your thoughts), episodic memory (trouble recalling what happened during the last week, forgetting details of conversations). Subscales are means of constituent items. Mood [Mental Health Inventory (MHI-5)^83^] was a covariate in MSCS analyses.

We collected MSCS and MHI-5 data from neurologically-healthy control respondents via anonymous electronic capture (REDCap); the link was sent to patients and employees who were asked to share it with acquaintances (at their discretion). Respondents aged ≥18 years provided age (5-year spans), sex, education and history of neurologic condition. We excluded data from those aged >65 years, with missing data or with a neurologic condition.

MSCS data were combined across clinical periods; patients were divided into subgroups of early relapsing-remitting, later-relapsing-remitting and secondary-progressive disease (as described earlier). We derived a comparison sample of 500 consecutive healthy respondents matched to patients in age, sex and education. Given expected demographic differences across multiple sclerosis subgroups, MSCS items were adjusted for age, sex and education [using a General Linear Model (GLM)]. Independent *t*-tests (equal variances not assumed, *P* adjusted for false discovery rate) assessed for differences in MSCS cognitive domains between patients and controls, and then across multiple sclerosis subgroups and controls. All analyses were repeated after adjusting MSCS for mood (MHI-5).

##### Historical comparisons

From August 2018 to September 2021, subjective cognition was additionally assessed in patients and control respondents with the Perceived Deficits Questionnaire (PDQ),^84^ a 20-item self-report inventory of difficulties with attention/executive function and memory. The original publication from 1990 reported subscale means for persons with multiple sclerosis (*n* = 1180) and controls (*n* = 200),^84^ allowing comparisons of patient-reported attention/executive (mean of attention/concentration and planning/organization subscales) and memory (mean of retrospective memory and prospective memory subscales) now versus 35 years ago, which is bolstered by contemporaneous controls for respective patient groups. One-sample *t*-tests assessed for differences in mean attention/executive and memory difficulties between patients now versus previously (1990), and control respondents now versus previously (1990). Independent *t*-tests derived effect sizes (*g*) for case-control differences in self-reported attention/executive and memory between current patients and current controls, which we compared to effect sizes for differences between previous patients and controls.^84^

### Ethical approval

The Institutional Review Board at the Icahn School of Medicine at Mount Sinai approved the retrospective chart review and determined that written informed consent was not required, and approved the RADIEMS project, for which all participants provided written informed consent.

## Results

### RADIEMS case-control cohort

#### Cross-sectional

Year 0 data were collected from 170 patients and 45 neurologically-healthy friends and non-first-degree relatives (Supplementary Table 1). There were no group differences in baseline characteristics except for a trending difference in estimated premorbid ability (WTAR) favouring controls (*P* = 0.10); raw scores were adjusted for WTAR (using GLM). As shown (Supplementary Fig. 1A and Supplementary Table 2), patients performed worse than controls on SDMT (*P* = 0.006, *g =* 0.451) and SRT-CLTR (*P* = 0.012, *g =* 0.426), but not on SCWT (*P* = 0.572, *g =* 0.081), SRT-LTS (*P* = 0.142, *g =* 0.247) or SRT-DR (*P* = 0.198, *g =* 0.200). The pattern of results was similar without adjusting for WTAR: SDMT (*P* < 0.001, *g =* 0.528), SCWT (*P* = 0.186, *g =* 0.189), SRT-LTS (*P* = 0.044, *g =* 0.327), SRT-CLTR (*P* = 0.003, *g =* 0.500), SRT-DR (*P* = 0.080, *g =* 0.261).

#### Longitudinal

Year 3 and Year 6 cognitive data were collected from 160 (94%) and 138 (81%) patients, respectively, with medians (IQRs) of 3.1 (3.0, 3.4) and 6.2 (6.0, 6.5) years since baseline (SRT missing for four patients at Year 6 due to time constraints). There were no differences in baseline characteristics between patients who did versus did not return for either follow-up (*P*’s > 0.10). From Year 0 to Year 3 (Supplementary Fig. 1B), patients showed a small increase in SDMT (*P* = 0.007, *d* = 0.215), but no change in SCWT (*P* = 0.702) or any SRT variable (*P*’s > 0.10). From Year 0 to Year 6 (Supplementary Fig. 1C), there were no changes in SDMT (*P* = 0.080, *d* = 0.150), SCWT (*P* = 0.343, *d* = 0.081), or SRT-DR (*P* = 0.155, 0.123), but there was a decline in SRT-CLTR (*P* = 0.002, *d* = 0.268), and a trend toward decline in SRT-LTS (*P* = 0.025, *d* = 0.196; raw scores are provided in Supplementary Table 2).

For controls, Year 0 to Year 3 attrition was higher (−33%) and strongly related to lower estimated premorbid ability (WTAR; *P* = 0.019, *g =* 0.789); therefore, data were not analysed [for transparency, cognitive change did not differ from patients on any task (*P*’s > 0.25), especially SDMT and SCWT (*P*’s > 0.75)].

#### Historical comparisons

Age, sex and time since diagnosis were similar for historical studies^48-51^ and RADIEMS (Table 1), but diagnostic criteria and treatment were more advanced in RADIEMS. Relative to historical studies, effect sizes for case-control differences within RADIEMS appeared slightly smaller for SDMT and SRT, but much smaller for Stroop. An additional historical report^85^ from 2001 reported a significant 14.9% decline in cognitive speed on Stroop over 2 years in untreated patients with early relapsing-remitting disease (*n* = 53) diagnosed using Poser 1983 criteria.^9^ In contrast, cognitive speed on Stroop did not change over 6 years in our cohort.

**Table 1:** Case-control differences in cognition within historical versus current cohorts of early relapsing-remitting multiple sclerosis

Study	Deloire *et al.*^49^		Olivares *et al.*^51^		Amato *et al.*^48^		Ruet *et al.*^50^		Summary of four prior studies^48-51^		RADIEMS cohort	
Group	RRMS	Control	RRMS	Control	RRMS	Control	RRMS	Control	RRMS	Control	RRMS	Control
Sample size, *n*	44	44	33	33	49	56	60	310	186	443	170	45
Age, mean (SD)	38.0 (9.5)	37.8 (10.8)	28.9 (7.8)	28.2 (6.5)	36.9 (8.9)	38.1 (9.0)	37.3 (9.9)	37.3 (9.9)^a^	35.9 (9.7)	36.2 (10.1)^b^	34.1 (7.4)	33.3 (7.6)
Sex, % female	68.2	68.2	75.8	75.8	77.6	64.3	81.7	81.7^a^	76.3	72.5^b^	66.5	64.4
Years diagnosed, mean (SD)	0.3 (0.3)	–	1.8 (1.8)	–	2.9 (1.7)	–	4.1 (3.0)	–	2.5 (2.5)	–	2.2 (1.4)	–
Diagnostic criteria	Poser 1983	–	Poser 1983	–	Poser 1983	–	Poser 1983	–	Poser 1983	–	McDonald 2010	–
Disease modifying therapy, % (*n*)												
Any therapy	31.8 (14)	–	30.3 (10)	–	0 (0)	–	88.3 (53)	–	41.4 (77)	–	92.9 (158)	–
S1P inhibitor, fumarates	0 (0)	–	0 (0)	–	0 (0)	–	NR	–	0 (0) or NR	–	53.5 (91)	–
Monoclonal antibody	0 (0)	–	0 (0)	–	0 (0)	–	NR	–	0 (0) or NR	–	21.2 (36)	–
Expanded Disability Status Scale	2.0^c^	–	1.6^d^	^–^	1.7^d^	^–^	1.5^c^	^–^	1.5–2.0^c^	–	1.0^c^	^–^
Effect size for case-control differences, *g* [95%CI]												
SDMT	1.445		0.661		0.265		0.597		0.679^e^		0.451 [0.120, 0.780]	
Stroop	0.793		0.558		0.054		0.503		0.449^e^		0.081 [−0.247, 0.408]	
SRT	0.521		0.333		0.313		0.323		0.367^e^		0.311 [−0.018, 0.639]	

Sample characteristics and effect sizes (Hedge’s *g*) for case-control performance differences are reported for four previous case-control studies^48-51^ of early relapsing-remitting multiple sclerosis (RRMS; summary statistics and weighted mean effect sizes across prior studies are presented in the ‘Summary of four prior studies’ column. Sample characteristics and case-control effect sizes (with 95% confidence intervals) are reported to our RADIEMS Cohort. Effect sizes for case-control differences in verbal memory [Selective Reminding Test (SRT)] were similar in RADIEMS [0.311 (−0.018, 0.639)] and prior studies (0.367), but the case-control difference in cognitive speed across prior studies (*g =* 0.449) was not observed in RADIEMS [*g =* 0.081 (−0.247, 0.408)]. Case-control differences on the Symbol Digit Modalities Test (SDMT) was observed in prior studies (0.679) and RADIEMS [0.451 (0.120, 0.780)]. SD = standard deviation.

^a^Age and sex were not reported for the control group, but authors stated that age and sex were matched to the RRMS sample.

^b^To avoid undue weighting of controls in Ruet *et al*.,^50^ sample size for controls in Ruet *et al.*^50^ was changed from 310 to 60 when deriving summary statistics across historical studies.

^c^Median.

^d^Mean.

^e^Weighted mean effect size.

### Mount Sinai Hospital cognitive screening cohort

#### Sample characterizations

Samples are characterized in Table 2. Data were captured for 642 patients during the Symbol Search period, including early relapsing-remitting (<5.0 years, *n* = 271), later relapsing-remitting (≥5.0 years, *n* = 281) and secondary-progressive (*n* = 90) disease (Supplementary Table 3). All patients completed Symbol Search, HVLT and PAL; completion of BTA (*n* = 566, 88%), LM (*n* = 545, 85%), WLG (*n* = 466, 73%) and Tower (*n* = 322, 50%) did not differ by demographics or disability. Age difference across disease subgroups was mitigated with age-adjusted normative scores. Secondary-progressive disease was associated with male sex, lower education and lower estimated premorbid ability; these were covariates in comparisons across subgroups. Disease duration did not differ between later relapsing-remitting and secondary-progressive subgroups. Data were captured for 123 and 239 consecutive patients for the Stroop and Trail-Making periods, respectively. MSCS was available for 97.7% (981 of 1004) of the combined clinical cohort; we derived a control respondent sample (*n* = 500) matched to patients in age, sex, and education. Mood (MHI-5) was better in controls [75.2 (74.0, 76.3)] than patients [71.0 (70.1, 71.8)].

**Table 2:** Clinical cohort characteristics

	Symbol Search period	Stroop period	Trail-Making period
Sample Size, *n*	642	123	239
Age, years, mean (SD)	42.8 (11.3)	41.9 (10.7)	40.0 (10.0)
Sex, *n* (%)			
Female	461 (71.8)	94 (76.4)	164 (68.6)
Male	181 (28.2)	29 (23.6)	75 (31.4)
Race and Ethnicity, *n* (%)			
American Indian	2 (0.3)	0 (0.0)	0 (0.0)
Asian	14 (2.2)	6 (4.9)	13 (5.4)
Black, (non-Latino/a)	128 (19.9)	31 (25.2)	36 (15.1)
Latino/a	124 (19.3)	21 (17.1)	50 (20.9)
White (non-Latino/a)	374 (58.3)	65 (52.8)	140 (58.6)
Literacy (WTAR, eFSIQ *z*), mean (SD)	0.29 (0.71)	0.35 (0.74)	0.22 (0.72)
Bachelor’s degree, *n* (%)	460 (71.7)	76 (61.8)	174 (72.8)
Disease Ccourse, *n* (%)			
Relapsing-remitting	552 (86.0)	109 (88.6)	218 (91.2)
Secondary-progressive	90 (14.0)	14 (11.4)	21 (8.8)
Years since diagnosis, median [IQR]	5.75 [2, 12]	6 [2, 12]	5 [1, 14]
Disease modifying therapy, *n* (%)			
None	94 (14.6)	12 (9.8)	19 (7.9)
Lower efficacy	113 (17.6)	5 (4.1)	18 (7.5)
Interferon β1a	16	1	3
Peginterferon β1a	3	1	1
Glatiramer acetate	65	0	6
Teriflunomide	29	3	6
Cellcept	0	0	2
Moderate efficacy	170 (26.5)	21 (17.1)	43 (18.0)
Dimethyl fumarate	105	6	14
Diroximel fumarate	21	9	10
Fingolimod	34	3	7
Siponimod	5	2	2
Ozanimod	5	1	10
High efficacy	265 (41.3)	85 (69.1)	159 (66.5)
Rituximab	12	1	3
Natalizumab	69	16	34
Alemtuzumab	0	0	1
Ocrelizumab	181	58	80
Ofatumumab	3	9	35
Ublituximab	0	0	4
Cladribine	0	1	2
Multiple Sclerosis Functional Composite			
Raw, median [IQR]			
SDMT	52 [45, 59]	52 [46, 59]	56 [49, 64]
NHPT	21.7 [19.3, 24.4]	21.1 [19.0, 23.6]	20.8 [18.9, 23.3]
T25FW	4.5 [4.1, 5.3]	4.5 [4.0, 5.1]	4.3 [3.9, 4.8]
T25FW cut-off, *n* (%)			
≥5.5s	140 (22.0)	21 (17.4)	22 (9.4)
≥6.0s	105 (16.5)	10 (8.3)	14 (6.0)
≥8.0s	36 (5.7)	8 (6.5)	8 (3.3)

eFSIQ = estimated Full Scale Intelligence Quotient; IQR = interquartile range; NHPT = Nine Hole Peg Test; SDMT = Symbol Digit Modalities Test; T25FW = Timed 25 Foot Walk; WTAR = Wechsler Test of Adult Reading.

#### Symbol search period

**Figure 3:**
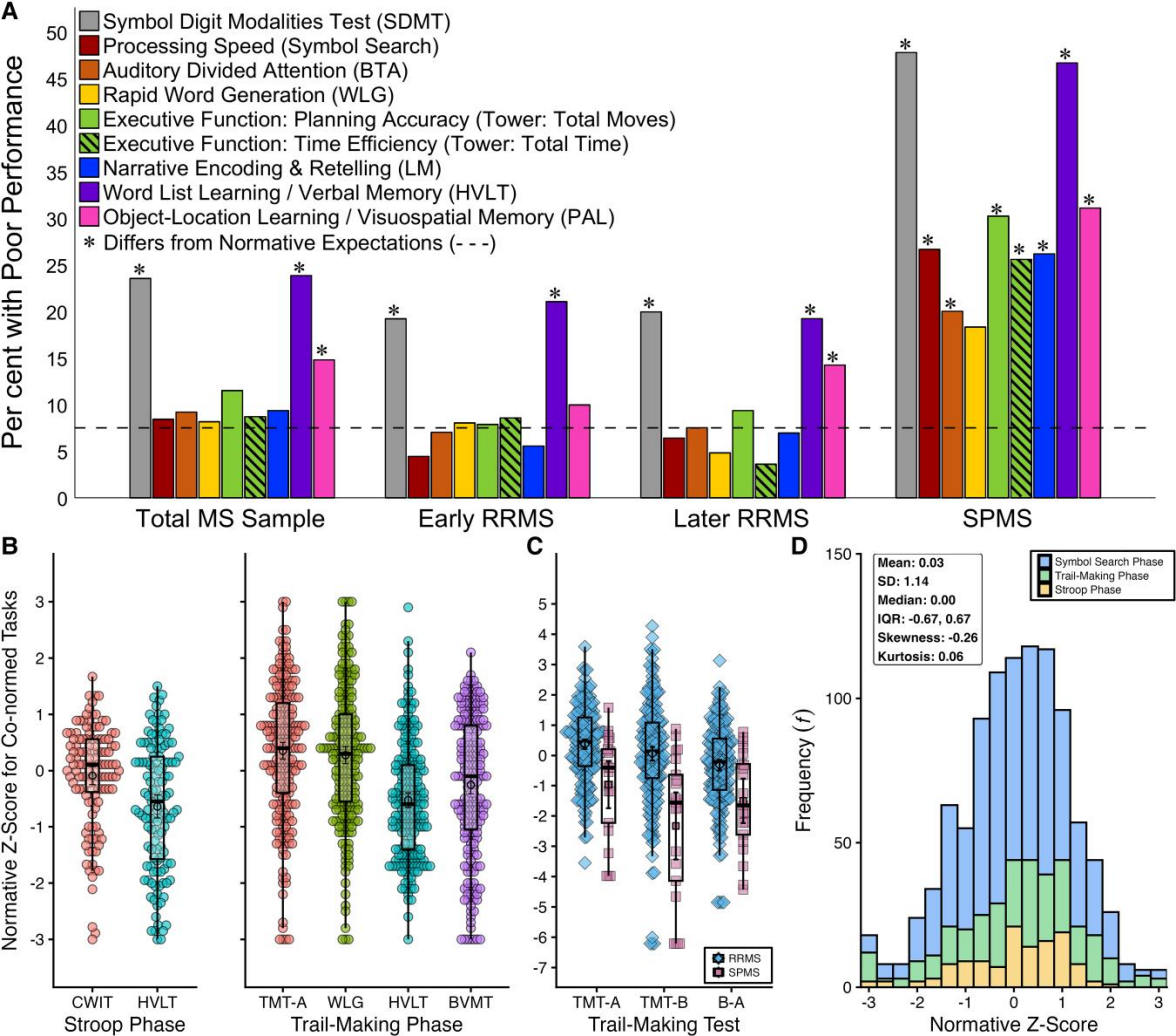
**Cognitive performance within the three clinical periods.** (**A**) Results for the Symbol Search period: rates of poor performance (≤7.5th percentile, indicated by dashed line) across cognitive tasks (colour-coded) for the total sample and for disease subgroups of early relapsing-remitting multiple sclerosis (Early RRMS), later RRMS, and secondary progressive multiple sclerosis (SPMS). An asterisk indicates a significant difference between the observed and expected (7.5%) rate of poor performance for each task. (**B**) All data for co-normative *z*-scores during the Stroop period (*left*) and Trail-Making period (*right*). Black circles with bars indicate means and 95% confidence intervals; box plots depict medians and interquartile ranges. (**C**) Co-normative^77^  *z*-scores separately for patients with RRMS versus SPMS across cognitive speed on Trail-Making Test part A (TMT-A), cognitive speed plus executive control on TMT-B, and executive control isolated from cognitive speed (B − A). Black diamonds (RRMS) and squares (SPMS) with bars indicate means and 95% confidence intervals; box plots depict medians and interquartile ranges. (**D**) Histogram showing the frequency of normative *z*-scores (in scaled score intervals) for cognitive speed tasks within the combined clinical cohort, colour-coded by period (i.e. test). Descriptive statistics in the box indicate close approximation to a normal distribution.

Rates of poor performance relative to normative expectations (7.5%) across tasks are presented for the total patient sample and disease subgroups (Fig. 3A and Supplementary Table 4). Within the total sample, rates of poor performance were elevated for SDMT, word-list memory (HVLT), and object-location memory (PAL), but not for cognitive speed (Symbol Search), auditory divided attention (BTA), rapid word generation (WLG), executive function (Tower) or narrative encoding (LM). For disease subgroups, rates of poor performance were elevated for only SDMT and HVLT in early relapsing-remitting disease, and only SDMT, HVLT, and PAL in later relapsing-remitting disease, but were elevated for all tasks except WLG in secondary-progressive disease. Rates of poor performance did not differ between early versus later relapsing-remitting disease on any task (*P*’s > 0.10), but were higher for secondary-progressive than later relapsing-remitting disease on every task (*P*’s ≤ 0.001).

##### Historical comparisons

As shown in Table 3, left), risk for poor Symbol Search performance in relapsing-remitting disease was 68.5% lower in our cohort (5.4% of sample, *n* = 30) than in the 2004 sample^4^ [15.4%; *P* < 0.001; odds ratio (OR) = 0.315 (0.179, 0.553)]; risk in secondary-progressive disease was 74.2% lower in our cohort (26.7%, *n* = 24) than the 2004 sample [58.5%; *P* < 0.001; OR = 0.258 (0.126, 0.530)]. In relapsing-remitting disease, there were large differences in normative *z*-scores favouring our cohort [0.13 (0.05, 0.22)] over samples reported^4^ in 2004 [mean difference (MD) = 0.75 (0.66, 0.84), *P* < 0.001, *g =* 0.73] and those reported^68^ in 2012 [MD = 0.88 (0.80, 0.97), *P* < 0.001, *g =* 0.86]. In contrast, there was only a small difference between our cohort and the more contemporaneous 2022 sample^69^ [MD = 0.20 (0.12, 0.29), *P* < 0.001, *g =* 0.20].

**Table 3:** Cognitive speed and self-reported cognitive deficits in historical versus current samples

	Symbol Search				Stroop paradigm			Self-reported cognitive difficulty (PDQ)			
Studies	DeLuca *et al.*^4^	Ryan *et al.*^68^	Manglani *et al.*^69^	Current sample	1987–99	2003–08	Current sample	Sullivan *et al.*^84^		Current sample	
Group	RRMS	RRMS	RRMS	RRMS	MS^a^	MS^a^	MS^a^	MS^a^	Control	MS^a^	Control
Sample size, *n*	162	40	87	552	216	248	123	1180	200	300	200
Age, mean (SD)	44.7 (9.0)	42.1 (9.9)	47.3 (7.9)	41.5 (11.0)	41.1 (9.5)	45.1 (9.7)	41.9 (10.7)	49 (NR)	Matched	49.2 (8.2)	49.3 (9.1)
Sex, % female	72.8	82.5	79.3	74.6	59.7	66.5	76.4	72.0	Matched	72.0	72.0
Years diagnosed, mean (SD)	5.1 (5.8)	8.2 (7.8)	10.4 (6.5)	6.7 (6.0)	9.2 (6.6)	9.3 (7.0)	7.2 (6.2)	11.3 (NR)	n/a	8.4 (5.7)	n/a
Diagnostic criteria	Poser 1983	McDonald 2001	NR	McDonald 2001–17	Poser 1983	Poser 1983 (1), McDonald 2001 (4)	McDonald 2001–17	NR	n/a	McDonald 2001–17	n/a
Symbol Search, normative *z*-score, mean (SD)	−0.62 (1.07)	−0.75 (1.06)	−0.07 (1.17)	0.13 (1.02)	–	–	–	–	–	–	–
Stroop, effect size for case-control differences, Hedge’s *g*	–	–	–	–	0.91	0.93	0.02	^–^	^–^	^–^	^–^
Self-reported cognitive deficits on PDQ, mean (SD)	–	–	–	–	–	–	–	–	–	–	–
Attention/Executive	–	–	–	–	–	–	–	1.70 (0.90)	1.15 (0.50)	1.52 (1.06)	1.16 (0.71)
Memory	–	–	–	–	–	–	–	1.40 (0.80)	0.95 (0.45)	1.41 (0.99)	0.97 (0.59)

Data from historical comparisons and current patients are shown for Symbol Search (*left*), the Stroop paradigm (*middle*), and self-reported cognitive difficulty assessed with the Perceived Deficits Questionnaire (PDQ; *right*). On the left, demographic information, clinical characteristics, and normative *z*-scores for Symbol Search among patients with relapsing-remitting multiple sclerosis (RRMS) are reported for three previous samples^4,68,69^ and our current sample; normative *z*-scores [mean (SD)] were below expectations in 2004 [−0.62 (1.07)] and 2012 [−0.75 (1.06)], but not in 2022 [−0.07 (1.17)] or currently [0.13 (1.02)]. In the centre, we present summary demographic information, clinical characteristics, and effect sizes for case-control differences for the Stroop colour-word condition across five studies from 1987 to 1999 and five studies from 2003 to 2008. Data for studies from 2003 to 2008 were presented in a meta-analysis.^67^ We reviewed studies^70-74^ from 1987 to 1999 and derived mean weighted effect sizes for case-control differences. Case-control differences between patients with multiple sclerosis and controls were large and comparable for articles from 1987 to 1999 (*g =* 0.91) and 2003 to 2008 (*g =* 0.93), but current patients did not differ from normative expectations (*g =* 0.02). On the right, we present demographic information, clinical characteristics, and self-reported attention/executive deficits and memory deficits (PDQ) for patients and controls reported in 1990,^84^ and age- and sex-matched current patients and controls. Subjective memory deficits [mean (SD)] were comparable between patients in the 1990 [1.40 (0.80)] and current [1.41 (0.99)] samples (*P* = 0.838, *g =* 0.01), but subjective attention/executive deficits were worse among patients in 1990 [1.70 (0.90)] than in the current sample [1.52 (1.06); *P* = 0.005, *g =* 0.17]. There were no differences in reported attention/executive deficits (*P* = 0.812, *g =* 0.02) or memory deficits (*P* = 0.701, *g =* 0.03) between control respondents in the 1990 versus current samples (affording confidence that samples are comparable). MS = multiple sclerosis; n/a = not applicable; NR = not reported.

^a^Mixed course.

#### Stroop period

Relative to co-normative data (Fig. 3B),^64^ cognitive speed on Stroop was normal [CWIT: −0.09 (−0.25, 0.07)] and better than verbal memory [HVLT, −0.64 (−0.85, −0.44); MD = 0.55 (0.34, 0.76), *P* < 0.001, *d* = 0.47]; within-subjects rate of poor performance (≤7.5th percentile) was much higher for verbal memory (26.8%, *n* = 33) than for cognitive speed (8.1%, *n* = 10; *P* < 0.001).

##### Historical comparisons

Case-control studies^5,70-76^ published from 1987 to 2008 (for review see Denney *et al*.^67^) reported a large cognitive speed deficit among patients on the Stroop colour-word task (*g*: 0.91 to 0.93; Table 3, middle). In contrast, cognitive speed in our cohort was normal, with no difference versus the healthy standardization sample for Stroop colour-word [normative *z*-score: 0.02 (−0.17, 0.20), *P* = 0.861, *d* = 0.02], word [*z* = 0.09 (−0.07, 0.24)], or colour [*z* = −0.10 (−0.26, 0.05); *P*’s > 0.10] trials, or the mean of all trials [*z* = 0.00 (−0.15, 0.14), *P* = 1.000].

#### Trail-Making period

As shown in Fig. 3B, relative to co-normative data,^62^ TMT-A was normal [0.35 (0.20, 0.50)] and better than HVLT-TL [−0.53 (−0.65, −0.40); MD = 0.88 (0.72, 1.03), *P* < 0.001, *d* = 0.70] and BVMT-TL [−0.26 (−0.41, −0.10); MD = 0.61(0.44, 0.78), *P* < 0.001, *d* = 0.47]. WLG was also normal [0.28 (0.13, 0.43)] and better than HVLT-TL [MD = 0.81 (0.66, 0.95), *P* < 0.001, *d* = 0.70] and BVMT-TL [MD = 0.53 (0.37, 0.71), *P* < 0.001, *d* = 0.40]. Likewise, within-subjects rates of poor performance (≤5th percentile) were higher for HVLT-TL (18.8%, *n* = 45) and BVMT-TL (17.2%, *n* = 41) than for TMT-A (5.0%, *n* = 12) and WLG (5.4%, *n* = 13; *P*’s < 0.001).

Normative^77^  *z*-score for TMT-B was normal [−0.15 (−0.38, 0.08)] but lower than TMT-A [0.26 (0.09, 0.41); MD = 0.40 (0.23, 0.58), *P* < 0.001, *d* = 0.30]. Normative *z*-scores were lower in secondary-progressive than relapsing-remitting disease (Fig. 3C) for TMT-A [MD = 1.34 (0.60, 2.09), *P* = 0.006, *g =* 1.09], TMT-B [MD = 2.39 (1.34, 3.45), *P* < 0.001, *g =* 1.35], and the contrast score isolating executive control [MD = 1.87 (1.02, 2.74), *P* = 0.003, *d* = 1.36], consistent with executive deficits in secondary-progressive disease reported above. (All TMT differences between disease courses remained when reanalysed with Mann-Whitney U-tests, *P’*s < 0.001.)

#### Combined clinical cohort

##### Cognitive speed

Normative *z*-scores for cognitive speed were combined across periods (*n* = 1004); performance in the total cohort approximates a normal distribution [Fig. 3D; median (IQR): 0.0 (−0.67, 0.67)], with no difference between performance by patients and healthy normative expectations [MD = 0.03 (−0.04, 0.10), *P* = 0.397, *d* = 0.03].

##### SDMT performance

Despite normal cognitive speed, SDMT was below expectations in our combined clinical cohort [raw: 52.6 (51.8, 53.3); normative *z*: −0.69 (−0.76, −0.62)]. We compared current performance to an influential 2006 article.^34^ Our sample was younger [42.1 (10.9) versus 45.4 (8.9)] with a more balanced sex ratio (71.6% versus 78.0% female); estimated premorbid ability was average [*z* = 0.33 (0.71)] and comparable to estimates for the 2006 sample [0.35 (0.57)].^78-81^ Blind to everything except age and sex, we derived a subsample of 800 patients exactly matching the 2006 sample for age and sex. SDMT among our patients [52.1 (51.2, 52.9)] was higher than the mean of 47.8 in 2006 [MD = 4.25 (3.43, 5.08), *P* < 0.001, *d* = 0.36]. Risk for poor SDMT performance (*z* < −1.5 versus 2006 control group) was lower now (37.6%) than in 2006 [51.9%; *P* < 0.001, OR = 0.559 (0.427, 0.733)].

#### Self-reported cognitive difficulty

**Figure 4:**
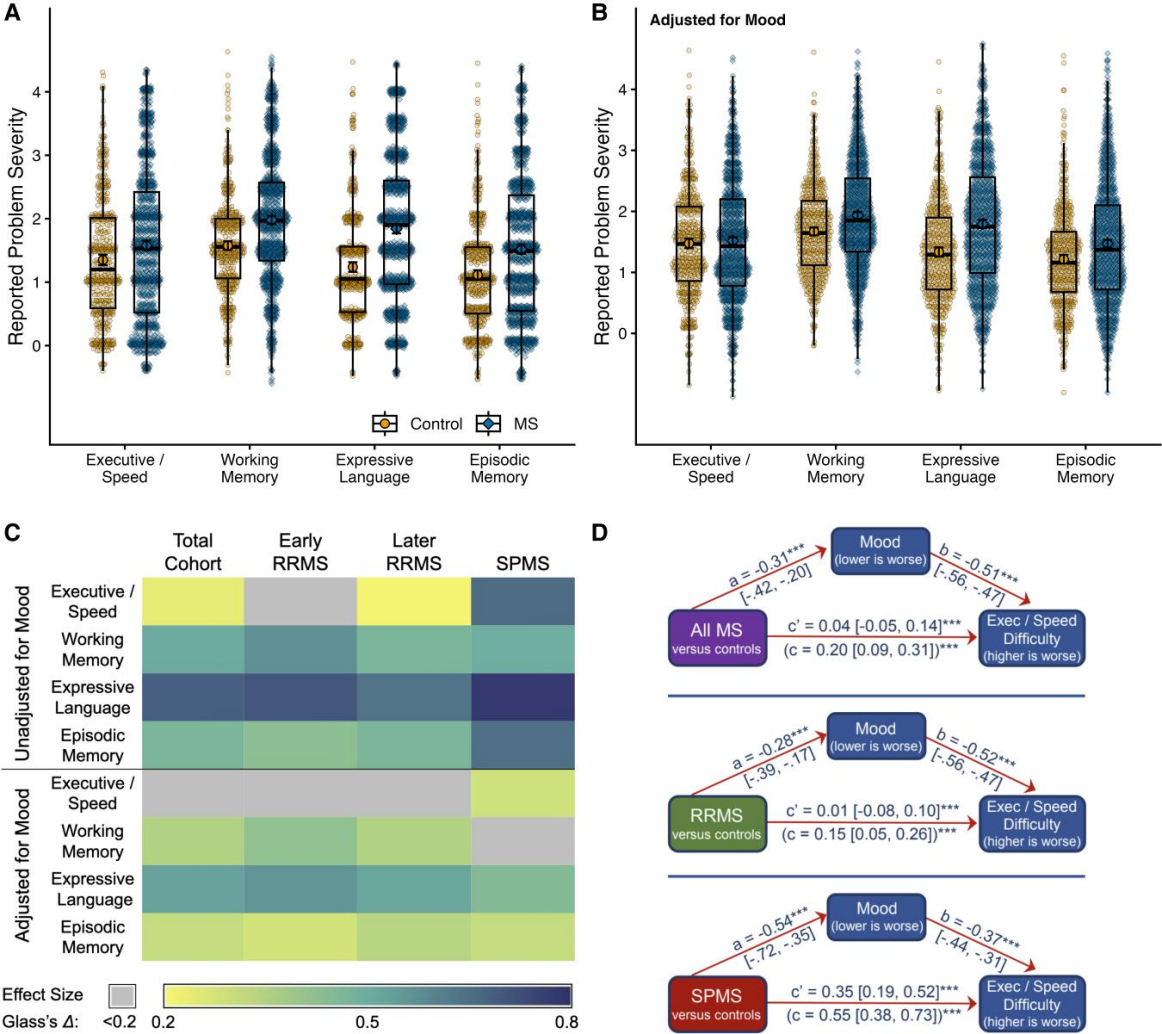
**Self-reported cognitive difficulty in patients versus controls.** (**A** and **B**) All data for self-reported cognitive difficulty across the four Multiple Sclerosis Cognitive Scale^82^ (MSCS) cognitive domains for patients and controls (**A**: adjusted for age, sex and education; **B**: adjusted for age, sex, education and mood). Black circles (controls) and diamonds (patients) with bars indicate means and 95% confidence intervals; box plots depict medians and interquartile ranges. (**C**) Heat map of effect sizes (Glass’s Δ) for differences in self-reported cognitive difficulties (four MSCS domains) between controls and the total patient sample, and also between controls and each disease subgroup: early relapsing-remitting multiple sclerosis (Early RRMS), Later RRMS, secondary-progressive multiple sclerosis (SPMS). Findings are presented adjusted for age, sex, and education (*top*), and adjusted for age, sex, education, and mood (*bottom*). Cells are colour-coded for effect size (key below). Specific effect sizes and 95% confidence intervals for all comparisons are provided in Supplementary Table 6. (**D**) presents mediation models evaluating whether mood mediates (explains) observed case-control differences in reported executive/speed difficulty (i.e. worse difficulty among patients). Models are presented separately for the total patient sample (*top*), RRMS (*middle*) and SPMS (*bottom*). As shown, only patients with SPMS reported elevated executive/speed deficits independent of mood.

Expressive language was the largest patient-reported deficit relative to neurologically-healthy controls (Fig. 4A), followed by working memory and episodic memory, and then a small difference in executive/speed. Adjusting for mood (Fig. 4B), patient-reported differences remained for expressive language, working memory and episodic memory, but not executive/speed. Findings were similar across disease subgroups; notable exception: executive/speed difficulty was greater in secondary-progressive disease, and remained after adjusting for mood (Fig. 4C and Supplementary Table 5). Unadjusted MSCS scores are provided in Supplementary Table 6. Formal mediation analyses (PROCESS 4.2 macro for SPSS, Fig. 4D) confirmed that mood fully mediated differences in reported executive/speed difficulty between controls and the total patient sample (80.0%) and relapsing-remitting disease (93.3%), but only partially explained differences in secondary-progressive disease (36.3%).

##### Historical comparisons

PDQ data were available for 453 consecutive patients and 350 control respondents; our cohort was younger [42.9 (11.4)] than the 1990 historical sample.^84^ We derived a subsample of 300 patients and 200 controls matching the historical sample for age and sex (Table 3, right). One-sample *t*-tests indicate less reported attention/executive difficulty among patients now than in 1990 [MD = −0.18 (−0.30, −0.05), *P* = 0.005, *g* = 0.17], but no difference in memory [MD = 0.01 (−0.10, 0.12), *P* = 0.838, *d* = 0.01]. Likewise, effect size for case-control differences in reported attention/executive difficulty is lower now [*g* = 0.39 (0.21, 0.57)] than previously (*g* = 0.64), but not different for memory difficulty now [*g* = 0.52 (0.34, 0.70)] than previously (*g =* 0.59). Confidence in comparisons to 35 years ago is bolstered by nearly identical self-reported difficulties by current and previous controls. Disease course was not reported in 1990, but time since diagnosis was longer. We trimmed our cohort to 253 patients diagnosed ≥5.0 years to match for years since diagnosis [11.3 (4.0)]; reported attention/executive difficulty was still less now [MD = −0.18 (−0.27, −0.04), *P* = 0.010, *d* = 0.16], with still no difference in reported memory deficits [MD = 0.01 (−0.11, 0.13), *P* = 0.926, *d* = 0.01].

#### Supplemental analysis

##### Verbal working memory

**Figure 5:**
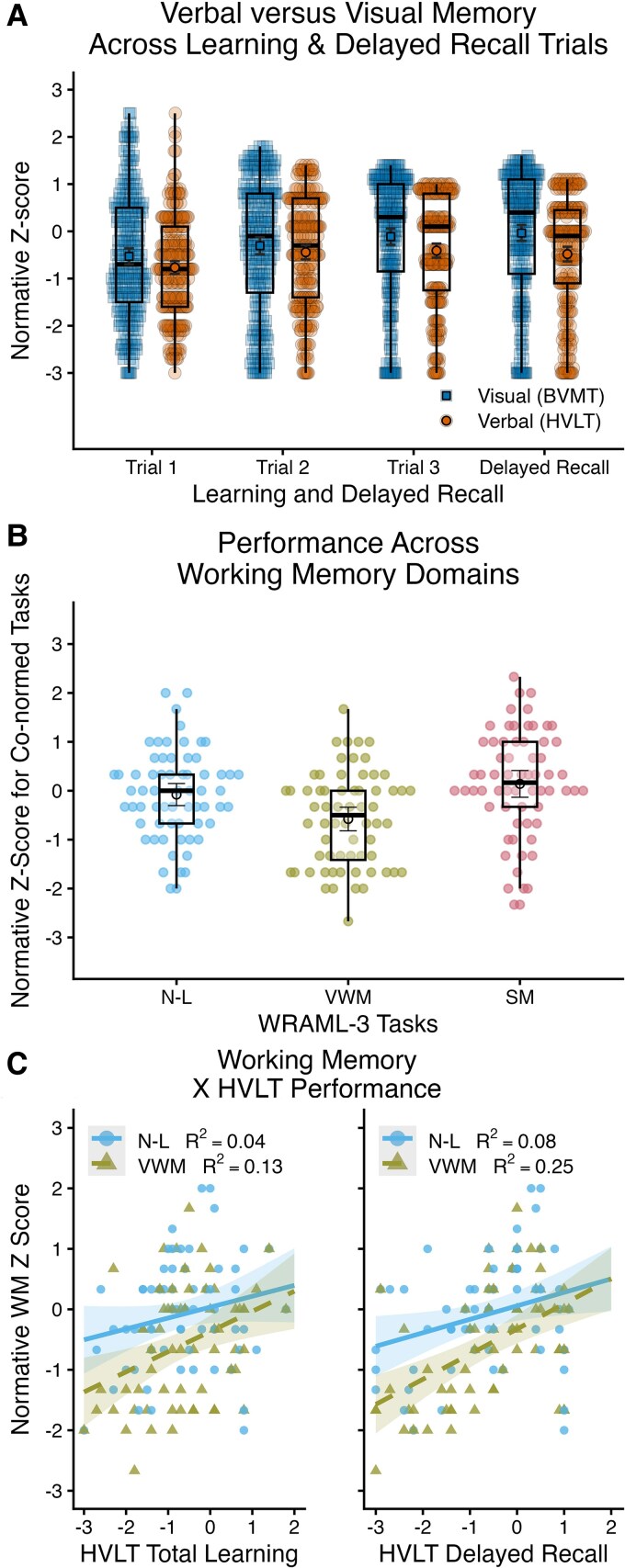
**Investigation of verbal working memory.** (**A**) displays normative *z*-scores for the Hopkins Verbal Learning Test (HVLT) and Brief Visuospatial Memory Test (BVMT) three learning trials (T1, T2, T3) and delayed recall (DR). Black circles with bars indicate means and 95% confidence intervals; box plots depict medians and interquartile ranges. (**B**) Normative *z*-scores across the Number-Letter (N-L), Verbal Working Memory (VMW), and Story Memory (SM) tasks of the Wide Range Assessment of Learning and Memory (WRAML-3). (**C**) Scatterplots for correlations between normative *z*-scores for N-L and VWM with normative *z*-scores for HVLT Total Learning (TL, *left*) and HVLT-DR (*right*).

Word-list learning was consistently weak across samples and appeared worse than visuospatial learning on PAL (Fig. 3A) and BVMT (Fig. 3B). Indeed, among patients from the Trail-Making period (*n* = 239), normative *z*-scores^57,61^ were lower for HVLT than BVMT on TL (*P* = 0.023, *d* = 0.152) and DR (*P* < 0.001, *d* = 0.368). Examined more closely, repeated-measures ANOVA (Bonferroni-corrected pairwise comparisons) revealed a trial (T1, T2, T3, DR) by task (HVLT, BVMT) interaction whereby performance did not differ between HVLT and BVMT for T1 or T2, but T3 and DR were greater for BVMT than HVLT (Fig. 5A). We explored verbal working memory as a potential contributor to word-list memory deficits. From November 2023 to April 2024, 64 consecutive patients with relapsing-remitting disease completed co-normed tasks^86^ requiring (i) repetition of number-letter strings of increasing length [Number-Letter (N-L)]; (ii) immediate retelling of short stories [Story Memory (SM)]; and (iii) holding a mixed set of aurally presented animals and non-animals in mind, then reciting the animals in size order, followed by reciting non-animals in any order [Verbal Working Memory (VWM)]. VWM requires brief maintenance of information (non-animals) within working memory while attention is focused elsewhere (i.e. ordering animals by size). As shown in Fig. 5B, co-normative *z*-scores were normal for N-L [−0.11 (−0.35, 0.12)] and SM [0.10 (−0.18, 0.38)]; however, VWM was below expectations [−0.62 (−0.86, −0.37)] and worse than N-L (*P* < 0.001, *d* = 0.589) and SM (*P* < 0.001, *d* = 0.712). Patients completed the HVLT, which was more related to performance on VWM than on the traditional span task (N-L, Fig. 5C).

## Discussion

Research on cognition in multiple sclerosis conducted three decades ago established a speed-centric model of cognitive dysfunction positing a core deficit in processing speed that leads to downstream deleterious effects on memory.^1-5^ Although still dominant,^7,8^ this model appears poorly suited for the current era: cognitive speed was normal across four independent samples of persons with relapse-onset multiple sclerosis diagnosed using McDonald criteria within the past 25 years and treated with modern therapies. Despite normal cognitive speed, memory difficulty remains prevalent. Our findings are bolstered by multiple replications, direct comparisons to results of several historical studies, use of co-normed tasks of cognitive speed and memory, evidence of normal and stable cognitive speed over 6 years in early relapsing-remitting disease, and alignment with patient-reported deficits in memory but not executive/speed in relapsing-remitting disease.

### Potential mechanisms of preserved cognitive speed

Preservation of cognitive speed among current patients is best explained by dramatic improvements in the diagnosis and treatment of multiple sclerosis over the past couple decades. Patients in historical samples were diagnosed when higher disease burden was required to meet diagnostic criteria, and before availability of therapies with moderate or high efficacy for preventing inflammatory lesion formation. Patients with unchecked disease accumulated large white matter lesion burden, axonal loss and cerebral atrophy with negative implications for cognitive processing (for a review see Rocca *et al*.^87^). Analyses of clinical trial data^88^ linked disease modifying therapies to preserved performance on task requiring attention, executive control, and rapid information processing;^89^ this protective effect was mediated through treatment-related protection against cerebral atrophy.^88^

Historical deficits in cognitive speed and attention/executive function are likely also attributable to differences in the neuroinflammatory milieu between current and historical samples, independently of structural changes (e.g. cerebral atrophy). Serial MRI studies of gadolinium-enhancing lesion formation in untreated relapsing-remitting disease revealed that clinical relapses represent only a small fraction of the total number of new enhancing lesions,^90-92^ and about 30% of patients with relapsing-remitting disease had gadolinium-enhancing lesions 1 year after initiating interferon-beta-1a treatment^93^ (although better than the rate of 42.3% among patients on placebo). Moreover, each new acute inflammatory event is followed by weeks of inflammatory and metabolic disturbance and recovery, and each new inflammatory lesion has a risk of persisting as an area of compartmentalized inflammation.^94-96^ Note also that interferon-beta treatments were associated with increased circulating interleukin-6^97^ explaining iatrogenic flu-like symptoms.^98^ Taken together, patients with multiple sclerosis in historical samples had a much greater burden of neuroinflammation and systemic inflammation than current patients, with probable negative effects on attention/executive function and cognitive efficiency. Inflammatory cytokines signal downregulation of dopamine in the ventral striatum, leading to reductions in the reward processing necessary for sustained effort and attention/executive function^99,100^; Treadway *et al*.^99^ posit that reduced reward processing is a biologically-adaptive mechanism for preserving resources in the context of sickness or injury (imagine your own attention and cognitive speed during a fever), but chronically induces ongoing anhedonia often experienced phenomenologically, and perceived clinically, as fatigue.^101^ Heitmann and colleagues^102^ further discuss these concepts in multiple sclerosis. Consistent with the expected relationship between disrupted reward processing and attention/executive function and cognitive efficiency,^100^ we and others have linked depression (especially anhedonia) in multiple sclerosis to worse cognitive efficiency, but not memory.^103-106^ In sum, worse disease-related structural damage (lesions, atrophy) and more severe neuroinflammatory burden are plausible independent (potentially synergistic) contributors to worse attention/executive function and cognitive speed in historical patient samples.

### Attention/executive dysfunction

Attention/executive dysfunction was observed in secondary-progressive, but not relapsing-remitting, disease; this aligns with differential patterns of cortical atrophy across disease courses. Cortical atrophy begins in temporal and parietal cortices in early relapsing-remitting disease, with notable sparing of dorsolateral prefrontal and medial prefrontal cortices^26,27^; this pattern is remarkably similar to normative regional cortical myelin density (high density in temporal and parietal cortices, low density in dorsolateral and medial prefrontal cortices),^107^ suggesting that cortical atrophy in relapsing-remitting disease may be driven by cortical demyelination. Consistent with a shift toward more predominant neurodegeneration, atrophy of prefrontal cortices in secondary-progressive disease^26,27^ aligns with executive/speed deficits. Some patients with relapsing-remitting disease reported executive dysfunction, but this was mediated through worse mood. We posit that dysfunctional reward processing may encumber attention/executive function in naturalistic settings with greater demands for initiation, planning, and inhibition of distractions, despite normal objective performance in the clinic.^108^ Management of mood may ameliorate real-life executive dysfunction for many patients.

### Working memory and episodic memory

Neuropsychology typically assesses working memory as maintenance and manipulation of information within the focus of active attention (e.g. simple span tasks^52^), but this appears normal in historical samples,^4,109^ and current patients (N-L, Fig. 5B). In contrast, patients historically^109,110^ and currently show difficulty on complex span tasks requiring short-term maintenance of information after attention momentarily shifts elsewhere (i.e. the VWM task). This reflects what our patients describe, for example, forgetting why one entered a room, losing one’s train of thought during conversations (an idea fades away before a break in the conversation to contribute; to compensate, some patients report interrupting to avoid losing their thought). Cognitive neuroscience posits that maintenance of information outside of attention is supported by transient changes in synaptic weighting of neurons encoding an engram, which facilitates subsequent reactivation of that engram.^111-115^ This is a promising framework for investigating memory deficits in multiple sclerosis; as discussed by Di Filippo and colleagues,^116^ inflammation-related disruptions of synaptic plasticity may explain memory dysfunction in multiple sclerosis. Notably, patients demonstrated normal encoding and immediate recall of highly-contextualized stories (LM, SM), but difficulty with poorly-contextualized word-list learning. Cognitive neuroscience views story encoding as a discourse processing task during which persons construct and maintain a cohesive mental model (‘situation model’) consisting of mutually-activating/mutually-cuing story elements^117,118^ that likely facilitate reactivation of stories (perhaps compensating for reduced synaptic plasticity). Indeed, during verbal paired associate learning, performance is comparable to controls when word-pairs are semantically-related (e.g. apple-worm), but worse than controls when unrelated (e.g. glasses-bus).^5,109^ We also observed worse word-list learning than visuospatial learning (Fig. 5A); mechanisms of this discrepancy are unclear, but highlight verbal working memory for further investigation. As illustrated (Fig. 5A), patients made word-list learning gains from weakest performance on the first trial, but they did not reach normative expectations by the third trial. Delayed recall was comparable to performance on the third learning trial, suggesting that patients do not lose information; instead, working memory during learning may be the memory-limiting factor.

### Expressive language

Expressive language difficulty (e.g. word-finding) is the largest reported cognitive deficit among current patients. This aligns with greater vulnerability of temporal-parietal regions to disease-related cortical atrophy and cortical lesion formation,^26-28^ but appears inconsistent with normal performance on traditional language tasks,^119,120^ including WLG herein. We have linked patient-reported word-finding difficulty to poor phonemic processing,^121^ and are developing more sensitive tasks to quantify word-finding,^122^ but our understanding and measurement of this prevalent patient-reported difficulty^120,123^ remains limited. Note, however, that greater difficulty with word-list than visuospatial learning appears to implicate language.

### Critique of the speed centric model

The speed-centric model remains supported by interpretation of poor SDMT as evidence of slow cognitive speed^29,124-126^; however, this contrasts with purposeful development of the SDMT as a multifarious screener to detect if any cognitive problem exists, not to isolate any specific deficit.^32,33^ Indeed, ample evidence indicates that cognitive functions other than speed contribute to SDMT in multiple sclerosis^34-39^ (see principal component analyses of the MACFIMS battery^34,36^). Moreover, we were unable to find any empirical evidence identifying slow cognitive speed as the mechanism underlying poor SDMT in multiple sclerosis. Experimentation requires that other variables are held constant (or isolated) to better understand a given effect; we essentially isolated the variable of speed by documenting normal performance on multiple more parsimonious cognitive speed metrics, thereby demonstrating that a process other than speed must explain poor SDMT in multiple sclerosis. Conceptually, speed is not itself a discrete cognitive function; instead, slow performance signals that a deficit exists in one or more underlying processes required by a task. Analogously, patients may demonstrate gait disturbance on the Timed 25 Foot Walk, but it would be incorrect (and unhelpful) to attribute slow walking to a primary deficit in walking speed; this is reductionist, and discourages investigation into actual underlying deficits (e.g. imbalance, spasticity) that could become targets of rehabilitation. Cognitive rehabilitation grounded in the speed-centric model has not developed effective treatments for memory dysfunction,^41^ with recent large randomized controlled trials of cognitive activities plus exercise (CogEx)^14^ and mixed amphetamine salts^15^ failing on primary outcomes of cognitive speed (*P*’s > 0.80, although measured with SDMT), and on secondary memory outcomes. Notably, the notion that slow speed negatively affects memory has been mostly hypothetical with little empirical support. Although a correlation of 0.44 between cognitive speed and verbal memory in multiple sclerosis has been proposed as evidence of a causal relationship,^127^ cognitive speed and verbal memory were correlated 0.45 in 60 398 healthy adults across 52 studies.^128^ Cognitive speed and memory are correlated in multiple sclerosis because they are correlated in everyone, likely mediated through generalized ability determined by genetic and early environmental factors.

### Clinical recommendations

Anecdotally, many of our recently diagnosed patients discover dire cognitive prognoses on the internet; we recommend against such searches, and educate patients that much information reflects a time before effective treatments. Patients encounter environmental challenges to working memory, including intermittent and unpredictable interruptions at work. Patients should be encouraged to minimize distractions, for example, designated times for checking e-mail, protected time for uninterrupted work, completion of one task before starting another. Case-control differences in reported executive/speed deficits were explained by worse mood in relapsing-remitting disease (Fig. 4D); patients who report distractibility, poor concentration, or executive dysfunction should be screened for depression (especially anhedonia). We previously observed that mood covaries with patient-reported executive dysfunction (but not other cognitive domains), with fewer executive deficits when mood improved.^104^ Management of mood may ameliorate executive deficits in everyday life. Importantly, as discussed,^108^ referrals to psychiatrists or psychotherapists often present a catch-22 because depression symptoms (anhedonia) make it harder for patients to clear the hurdles of obtaining mental health treatment (especially in the USA); remedies may include treatment of depression by neurologists, or use of social work to close the loop on referrals to mental health professionals.

## Conclusion

Cognition in multiple sclerosis is different within the modern diagnostic and disease modifying treatment era. Cognitive speed is normal despite memory difficulty, and patients report deficits in word-finding, working memory and episodic memory, but not executive/speed independent of mood. The field requires a more translational and multidisciplinary approach to cognitive research that brings together all stakeholders and areas of relevant expertise, including clinical neurologists, neuropsychologists, cognitive neuroscientists, rehabilitation specialists and experts in multiple sclerosis neuropathology and neuroimmunology, as well as experts in other diseases with potentially overlapping mechanisms of cognitive disruption. We must incorporate perspectives of patients, who are the only true experts on the reality of living with multiple sclerosis, especially regarding symptoms such as cognitive change that are often invisible to others but impactful for the individual.^129^ Current absence of a translational and multidisciplinary approach is evident by (i) the dearth of research on word-finding difficulty despite patients reporting such difficulty to clinical neurologists for years; (ii) articles on neuropsychological results without biologically-informed, disease-related explanations; (iii) correlations between psychometric test results and MRI markers of disease burden without a cognitive neuroscientific theoretical model to refute, support, and/or modify; and (iv) trials of cognitive interventions without evidence of biological plausibility or real-world applicability. For perspective, drug development begins with basic scientists tailoring agents to underlying mechanisms of disease; in cognition, the fields of cognitive neuroscience and cognitive psychology are those basic science laboratories that we have largely bypassed in our pursuits. A translational and multidisciplinary approach will advance our understanding of cognitive dysfunction in multiple sclerosis, and propel us toward effective treatments for our patients.

## Supplementary Material

awaf446_Supplementary_Data

## Data Availability

Anonymized data not published within this article will be made available by request from any qualified investigator, as permitted according to institutional regulations.
