# Supplementary material for: Cognition in multiple sclerosis within the modern diagnostic and treatment era
Source: Brain. 2025 Nov 26;149(5):1732–49. doi: 10.1093/brain/awaf446 (PMC13140566; doi:10.1093/brain/awaf446)

Supplementary Table 1. RADIEMS Baseline Characteristics

Baseline (Year-0) demographic and clinical characteristics are presented for RADIEMS participants with relapsing-remitting multiple sclerosis (RRMS, n=170), and demographic characteristics for neurologically-healthy friends and non-first degree relatives of patients (n=45). Data were additionally collected from five colleagues serving as controls; however, these data are excluded from current and all future analyses due to a high likelihood of bias (i.e., all five with doctoral degrees; mean literacy >80th percentile).

	**RRMS**	**Controls**
Sample Size	170	45
Age (years), mean [SD]	34.1 [7.4]	33.3 [7.6]
Sex, N [%]		
Female	113 [66.5]	29 [64.4]
Male	57 [33.5]	19 [35.6]
Race and Ethnicity, N [%]		
American Indian	0 [0]	0 [0]
Asian	7 [4.1]	3 [6.7]
Black, (non-Latino/a)	24 [14.1]	7 [15.6]
Latino/a	39 [22.9]	3 [6.7]
White (non-Latino/a)	100 [58.8]	32 [71.1]
WTAR-estimated FSIQ, z-score, mean [sd]	0.54 [0.60]	0.70 [0.69]
Bachelor's degree, N [%]	133 [78.2]	45 [88.9]
Years since multiple sclerosis diagnosis, median [IQR]	1.9 [0.9, 3.3]	
Disease Modifying Therapy, N [%]		
None	12 [7.1]	
Platform injectables	29 [17.1]	
Teriflunomide	2 [1.2]	
S1p modulators, fumarates	91 [53.5]	
Monoclonal antibodies	36 [21.2]	
Expanded Disability Status Scale (EDSS), median [IQR]	1.0 [0.0, 1.5]	
Multiple Sclerosis Functional Composite, raw, median		
Symbol Digit Modalities Test	56 [49, 65]	63 [58.5, 68]
Nine Hole Peg Test	19.1 [18.1,	18.3 [16.8,
Timed 25 Foot Walk	4.1 [3.7, 4.4]	n/a

Supplementary Table 2. RADIEMS Cognitive Performance

Means and standard deviations are presented for baseline (Year-0) literacy-adjusted raw scores for patients and controls, and Year-3 – Year-0 change scores and Year-6 – Year-0 change scores for patients. See Supplementary Figure 1 for data presentation as dot plots.

	Mean [SD]	
	**RRMS**	**Controls**
**Year-0 Raw Scores**		
Symbol Digit Modalities Test	56.8 [10.1]	61.3 [9.3]
Stroop Color-Word Test, Color-Word	47.6 [9.7]	48.3 [7.3]
Selective Reminding Test		
Long-Term Storage	50.0 [10.5]	52.6 [10.5]
Consistent Long-Term Retrieval	39.6 [13.2]	45.3 [13.1]
Delayed Recall	8.69 [2.57]	9.20 [2.24]
**Year-3 – Year 0 Change Scores**		
Symbol Digit Modalities Test	1.67 [7.75]	
Stroop Color-Word Test, Color-Word	0.19 [6.18]	
Selective Reminding Test		
Long-Term Storage	-1.24 [10.04]	
Consistent Long-Term Retrieval	-0.09 [12.58]	
Delayed Recall	-0.02 [2.00]	
**Year-6 – Year 0 Change Scores**		
Symbol Digit Modalities Test	1.17 [7.83]	
Stroop Color-Word Test, Color-Word	0.61 [7.51]	
Selective Reminding Test		
Long-Term Storage	-2.30 [11.73]	
Consistent Long-Term Retrieval	-3.59 [13.37]	
Delayed Recall	-0.29 [2.36]	

Supplementary Table 3. Characteristics for Disease Subgroups of Symbol Search Period
Demographic information is presented separately for patients with early relapsing-remitting multiple sclerosis (Early RRMS), Later RRMS, and secondary-progressive MS (SPMS) included within the Symbol Search period. Abbreviations: Estimated Full Scale IQ (eFSIQ), Wechsler Test of Adult Reading (WTAR)

	**Early RRMS**	**Later RRMS**	**SPMS**
Sample Size	271	281	90
Age (years), mean [SD]	37.4 [10.3]	45.4 [10.3]	51.1 [8.9]
Sex, N [%]			
Women	193 [71.2]	219 [77.9]	49 [54.4]
Men	78 [28.8]	62 [22.1]	41 [45.6]
Race and Ethnicity, N [%]			
American Indian	1 [0.4]	0 [0.0]	1 [1.1]
Asian	5 [1.8]	8 [2.8]	1 [1.1]
Black, (non-Latino/a)	60 [22.1]	49 [17.4]	19 [21.1]
Latino/a	54 [19.9]	52 [18.5]	18 [20.0]
White (non-Latino/a)	151 [55.7]	172 [61.2]	51 [56.7]
Estimated Premorbid Cognition / Literacy (WTAR, eFSIQ z), mean [sd]	0.27 [0.70]	0.36 [0.69]	0.10 [0.79]
Bachelor’s degree, N [%]	192 [70.8]	211 [75.1]	57 [63.3]
Years since multiple sclerosis diagnosis, median [IQR]	1 [1, 3]	11 [8, 14]	10 [6, 14]

Supplementary Table 4. Rates of Poor Performance and Odds Ratios Relative to Normative Expectations

Percentages of patients with poor performance on each task within the total patient sample and within subsamples (Early RRMS, Later RRMS, SPMS) are presented in the left panel. Odds ratios indicating risk for poor performance relative to normative expectations (7.5%) are presented in the right panel. Bold font indicates significant results adjusting for multiple comparisons.

Performance Metrics	Percent [N] with Poor Performance				Observed vs Expected Rate of Poor Performance: Odds Ratio [95%CI]			
	Total Sample	Early RRMS	Later RRMS	SPMS	Total Sample	Early RRMS	Later RRMS	SPMS
SDMT	**23.5 [151 of 642]**	**19.2 [52 of 271]**	**19.9 [56 of 281]**	**47.8 [43 of 90]**	**3.81 [2.69, 5.38]**	**2.98 [1.73, 5.15]**	**3.08 [1.81, 5.25]**	**10.85 [4.52, 26.03]**
Symbol Search	8.4 [54 of 642]	4.4 [12 of 271]	6.4 [18 of 281]	**26.7 [24 of 90]**	1.14 [0.76, 1.70]	0.58 [0.28, 1.21]	0.85 [0.44, 1.63]	**4.31 [1.75, 10.63]**
BTA	9.0 [51 of 566]	7.9 [18 of 228]	6.3 [16 of 253]	**20.0 [17 of 85]**	1.24 [0.81, 1.89]	1.06 [0.53, 2.12]	0.83 [0.42, 1.66]	**2.79 [1.09, 7.12]**
WLG	8.2 [38 of 466]	8.0 [15 of 187]	4.8 [10 of 208]	18.3 [13 of 71]	1.09 [0.68, 1.76]	1.08 [0.51, 2.30]	0.61 [0.27, 1.37]	2.96 [0.995, 8.80]
Tower: Moves	11.5 [37 of 322]	7.9 [11 of 140]	9.4 [13 of 139]	**30.2 [13 of 43]**	1.61 [0.94, 2.76]	1.00 [0.42, 2.39]	1.33 [0.56, 3.15]	**5.78 [1.51, 22.1]**
Tower: Time	8.7 [28 of 322]	8.6 [12 of 140]	3.6 [5 of 139]	**25.6 [11 of 43]**	1.18 [0.67, 2.09]	1.10 [0.47, 2.58]	0.48 [0.16, 1.45]	**4.58 [1.18, 17.83]**
LM	9.4 [51 of 545]	5.6 [12 of 216]	6.9 [17 of 245]	**26.2 [22 of 84]**	1.27 [0.83, 1.95]	0.74 [0.34, 1.59]	0.94 [0.47, 1.87]	**3.90 [1.57, 9.74]**
HVLT-R	**23.8 [153 of 642]**	**21.0 [57 of 271]**	**19.2 [54 of 281]**	**46.7 [42 of 90]**	**3.87 [2.74, 5.47]**	**3.34 [1.95, 5.74]**	**2.95 [1.73, 5.03]**	**10.38 [4.32, 24.90]**
PAL	**14.8 [95 of 642]**	10.0 [27 of 271]	**14.2 [40 of 281]**	**31.1 [28 of 90]**	**2.15 [1.49, 3.10]**	1.39 [0.76, 2.54]	**2.06 [1.18, 3.59]**	**5.36 [2.20, 13.01]**

Supplementary Table 5. Effect sizes for Differences between Persons with Multiple Sclerosis and Control Respondents

Table presents effect sizes (Glass's Δ [95% confidence interval]) for differences in self-reported cognitive difficulties (four MSCS domains) between controls and the total patient sample, and also between controls and each disease subgroup: early relapsing-remitting multiple sclerosis (Early RRMS), Later RRMS, secondary-progressive multiple sclerosis (SPMS). Findings are presented adjusted for age, sex, and education (upper), and adjusted for age, sex, education, and mood (lower). Adjusting for false discovery rate, all comparisons are statistically significant expect for those in italics font.

Adjusted for Mood		MSCS Domain	Total Cohort	Early RRMS	Later RRMS	SPMS
	No	**Executive / Speed**	.24<br>[.13, .35]	*.15*<br>*[.02, .28]*	.21<br>[.08, .34]	.66<br>[.45, .86]
		**Working Memory**	.51<br>[.40, .63]	.57<br>[.43, .70]	.47<br>[.34, .60]	.50<br>[.29, .70]
		**Expressive Language**	.69<br>[.57, .80]	.71<br>[.58, .85]	.64<br>[.51, .78]	.78<br>[.57, .98]
		**Episodic Memory**	.47<br>[.36, .59]	.43<br>[.30, .57]	.47<br>[.33, .60]	.65<br>[.45, .86]
	Yes	**Executive / Speed**	*.05*<br>*[-.06, .15]*	*-.04*<br>*[-.17, .09]*	*.07*<br>*[-.06, .19]*	.29<br>[.08, .49]
		**Working Memory**	.35<br>[.24, .46]	.42<br>[.28, .55]	.35<br>[.22, .48]	*.15*<br>*[-.05, .35]*
		**Expressive Language**	.53<br>[.41, .64]	.56<br>[.42, .69]	.52<br>[.38, .65]	.45<br>[.25, .66]
		**Episodic Memory**	.31<br>[.20, .42]	.28<br>[.14, .41]	.34<br>[.21, .47]	.31<br>[.11, .52]

Supplementary Table 6. Unadjusted Means for MSCS Items and Composites

Unadjusted means, standard deviations (in parentheses), and 95% confidence intervals (in brackets) are presented for composites and individual items of the Multiple Sclerosis Cognitive Scale (MSCS); these are reported for control respondents (n=500) and the total multiple sclerosis (MS) sample (n=981), as well as patient subgroups with early relapsing-remitting MS (Early RRMS, n=426), Later RRMS (n=438), and secondary-progressive MS (SPMS, n=117).

	Mean (SD) [95% CI] (Unadjusted)				
**MSCS Items and Composites**	**Controls**	**Total MS**	**Early RRMS**	**Later RRMS**	**SPMS**
**Executive Function**	1.35 (0.97) [1.26, 1.44]	1.58 (1.23) [1.50, 1.65]	1.51 (1.22) [1.40, 1.63]	1.54 (1.26) [1.43, 1.66]	1.95 (1.13) [1.74, 2.15]
trouble getting started, even if you had lots to do	1.44 (1.11) [1.34, 1.54]	1.65 (1.31) [1.57, 1.73]	1.61(1.30) [1.49, 1.74]	1.62 (1.33) [1.49, 1.74]	1.90 (1.27) [1.67, 2.12]
taking a long time to finish things	1.25 (1.00) [1.17, 1.34]	1.51 (1.31) [1.43, 1.59]	1.42 (1.29) [1.29, 1.54]	1.47 (1.33) [1.34, 1.59]	2.00 (1.18) [1.79, 2.21]
**Working Memory**	1.58 (0.82) [1.51, 1.65]	1.98 (1.08) [1.91, 2.05]	1.99 (1.07) [1.89, 2.09]	1.97 (1.10) [1.87, 2.07]	2.00 (1.04) [1.81, 2.19]
losing your train of thought	1.75 (0.89) [1.67, 1.82]	2.10 (1.10) [2.04, 2.17]	2.12 (1.08) [2.02, 2.22]	2.09 (1.12) [1.99, 2.19]	2.12 (1.08) [1.92, 2.32]
forgetting what you came into the room for	1.40 (0.95) [1.32, 1.49]	1.85 (1.22) [1.78, 1.93]	1.85 (1.26) [1.73, 1.97]	1.85 (1.21) [1.74, 1.97]	1.88 (1.13) [1.68, 2.09]
**Expressive Language**	1.24 (0.90) [1.16, 1.32]	1.84 (1.21) [1.77, 1.92]	1.86 (1.20) [1.75, 1.97]	1.81 (1.24) [1.69, 1.92]	1.92 (1.13) [1.71, 2.12]
having a word ‘on the tip of your tongue’ but difficulty getting it out	1.41 (1.00) [1.32, 1.50]	2.03 (1.27) [1.95, 2.11]	2.04 (1.28) [1.92, 2.16]	2.01 (1.28) [1.89, 2.13]	2.09 (1.23) [1.87, 2.32]
having a sense of what you want to say, but trouble clearly expressing your thoughts	1.07 (1.00) [0.98, 1.15]	1.65 (1.32) [1.57, 1.74]	1.68 (1.30) [1.55, 1.80]	1.60 (1.36) [1.47, 1.72]	1.74 (1.21) [1.52, 1.96]
**Episodic Memory**	1.12 (0.87) [1.05, 1.20]	1.52 (1.18) [1.45, 1.60]	1.48 (1.17) [1.37, 1.59]	1.51(1.19) [1.41, 1.62]	1.71 (1.18) [1.50, 1.92]
forgetting details of a recent conversation	1.18 (0.94) [1.10, 1.26]	1.62 (1.25) [1.54, 1.69]	1.59 (1.25) [1.48, 1.72]	1.58 (1.26) [1.47, 1.70]	1.84 (1.22) [1.62, 2.05]
trouble recalling what happened during the last week	1.05 (0.99) [0.97, 1.14]	1.42 (1.27) [1.35, 1.50]	1.36 (1.25) [1.24, 1.48]	1.45 (1.28) [1.33, 1.57]	1.57 (1.28) [1.34, 1.80]

Supplementary Figure 1. Cognition in Early Relapsing-Remitting Disease: RADIEMS Cohort.

Panel A illustrates differences between patients and controls at Year 0 (baseline) on the Symbol Digit Modalities Test (SDMT), Stroop Color-Word Test (SCWT), and the Selective Reminding Test (SRT: long-term storage [LTS], consistent long-term retrieval [CLTR], delayed recall [DR]); values are z-scores derived from means and standard deviations of controls. Panels B and C presents raw score change on SDMT and SCWT for patients from Year 0 to Year 3 (n=160), and from Year 0 to Year 6 (n=137), respectively. See Supplementary Table 2 descriptive statistics.

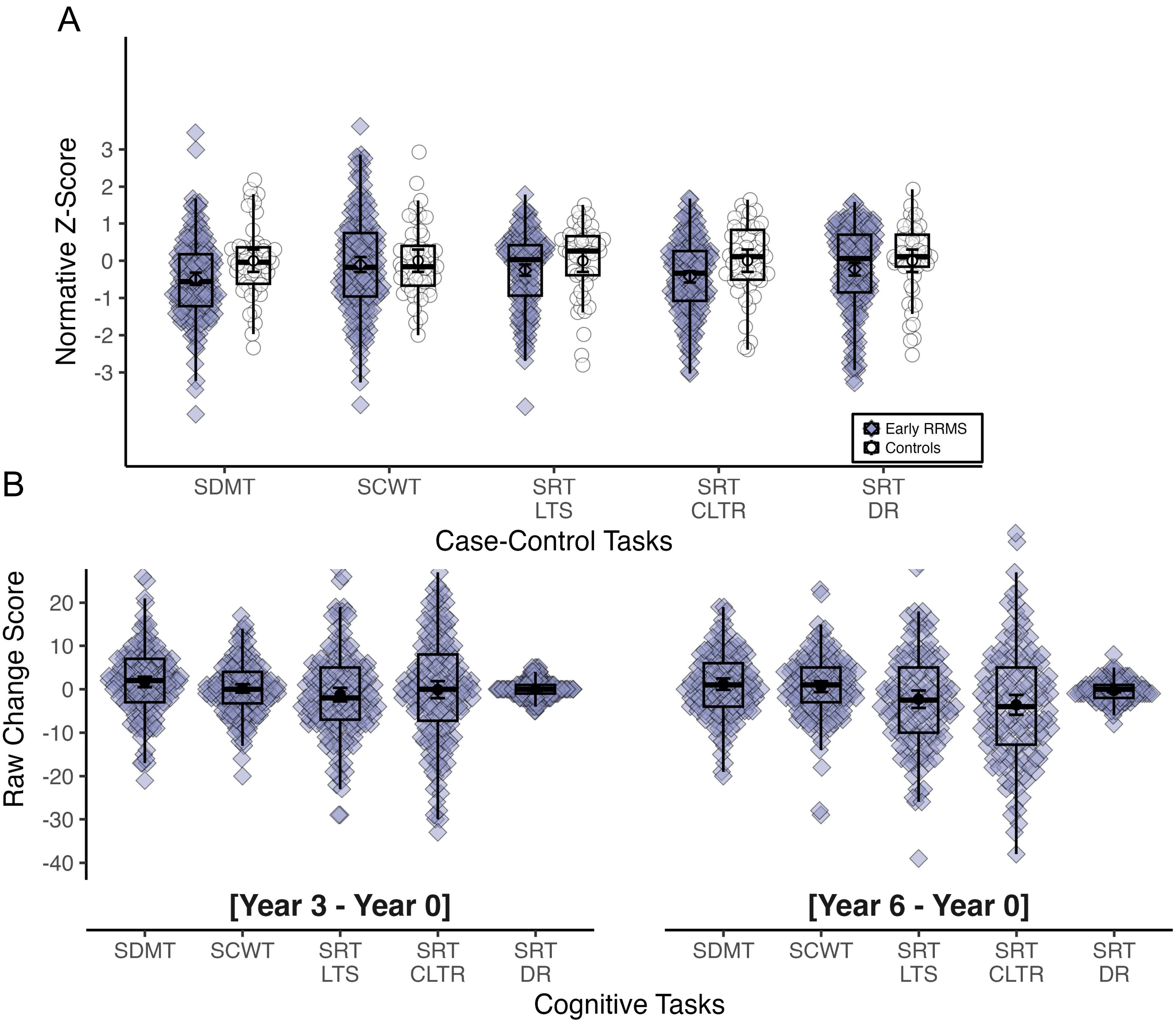

Supplement: awaf446_Supplementary_Data [file awaf446_supplementary_data.pdf]
